# *Starmerella bombicola *influences the metabolism of *Saccharomyces cerevisiae *at pyruvate decarboxylase and alcohol dehydrogenase level during mixed wine fermentation

**DOI:** 10.1186/1475-2859-11-18

**Published:** 2012-02-03

**Authors:** Vesna Milanovic, Maurizio Ciani, Lucia Oro, Francesca Comitini

**Affiliations:** 1 Dipartimento Scienze della Vita e dell'Ambiente, Università Politecnica delle Marche, 60121 Ancona, Italy

**Keywords:** Multistarter fermentation, *Saccharomyces cerevisiae*, *Starmerella bombicola*, Immobilization, Real-time RT-PCR

## Abstract

**Background:**

The use of a multistarter fermentation process with *Saccharomyces cerevisiae *and non-*Saccharomyces *wine yeasts has been proposed to simulate natural must fermentation and to confer greater complexity and specificity to wine. In this context, the combined use of *S. cerevisiae *and immobilized *Starmerella bombicola *cells (formerly *Candida stellata*) was assayed to enhance glycerol concentration, reduce ethanol content and to improve the analytical composition of wine. In order to investigate yeast metabolic interaction during controlled mixed fermentation and to evaluate the influence of *S. bombicola *on *S. cerevisiae*, the gene expression and enzymatic activity of two key enzymes of the alcoholic fermentation pathway such as pyruvate decarboxylase (Pdc1) and alcohol dehydrogenase (Adh1) were studied.

**Results:**

The presence of *S. bombicola *immobilized cells in a mixed fermentation trial confirmed an increase in fermentation rate, a combined consumption of glucose and fructose, an increase in glycerol and a reduction in the production of ethanol as well as a modification in the fermentation of by products. The alcoholic fermentation of *S. cerevisiae *was also influenced by *S. bombicola *immobilized cells. Indeed, Pdc1 activity in mixed fermentation was lower than that exhibited in pure culture while Adh1 activity showed an opposite behavior. The expression of both *PDC1 *and *ADH1 *genes was highly induced at the initial phase of fermentation. The expression level of *PDC1 *at the end of fermentation was much higher in pure culture while *ADH1 *level was similar in both pure and mixed fermentations.

**Conclusion:**

In mixed fermentation, *S. bombicola *immobilized cells greatly affected the fermentation behavior of *S. cerevisiae *and the analytical composition of wine. The influence of *S. bombicola *on *S. cerevisiae *was not limited to a simple additive contribution. Indeed, its presence caused metabolic modifications during *S. cerevisiae *fermentation causing variation in the gene expression and enzymatic activity of alcohol deydrogenase and pyruvate decarboxilase.

## Background

Wine fermentation is a complex process in which *Saccharomyces *and non-*Saccharomyces *yeasts can coexist and positively interact [1-7]. The control of spontaneous microflora involved during the winemaking process and the use of the inoculum of selected *S. cerevisiae *strains were considered to be fundamental steps to improve wine quality [8,9]. The use of a multistarter fermentation process with *S. cerevisiae *and non-*Saccharomyces *wine yeasts has been proposed to simulate natural must fermentation and to confer greater complexity and specificity to wine. The advantage of this process is to simulate a spontaneous process avoiding the risks of stuck fermentation [2,10-13]. Furthermore, non-*Saccharomyces *wine strains could have some specific enological characteristics that are absent in *S. cerevisiae *species, leading to combined, synergic and/or additive effects on the final wine [3,7,14-17].

In general, during a multistarter process, different microbial species live together, and the biotransformation of the nutritional sources is affected not only by the behavior of each microorganism but also by the interactions between different strains. In order to study this interaction, the hardest goal is to monitor the process which tries to understand the involvement of each individual yeast strain or its combined synergistic effect. In typical mixed-culture assays, the routine procedures used for tracking each strain are time-consuming, difficult and expensive [18]. For this reason, in order to monitor the behavior of each yeast during a multistarter experiment, some methods were based on cell separation by means of a porous membrane, while others were based on the cell immobilization technique [19,20].

In this work, we studied these interactions during the mixed fermentation of *S. cerevisiae *starter strain and a non-*Saccharomyces *enological yeast species, focusing our attention on *S. cerevisiae *during the most limiting steps of alcoholic fermentation: the decarboxylation of pyruvate to acetaldehyde and its subsequent reduction to ethanol. These steps are regulated by the expression of *ADH *and *PDC *genes. During *S. cerevisiae *growth on fermentable carbon sources, six *PDC *genes were identified out of which three structural genes (*PDC1, PDC5 *and *PDC6*) were encoded for active Pdc enzymes, independently [21]. These enzymes catalyze an irreversible reaction in which pyruvate is decarboxylated to acetaldehyde. Pdc1 is the predominant isoenzyme form (performing 80-90% of the activity in wild type cells). The regulatory genes *PDC2, PDC3 *and *PDC4 *encode proteins that are probably involved in the regulation of *PDC1 *and *PDC5 *expression. On the other hand, in *S. cerevisiae *there are five genes that encode alcohol dehydrogenases involved in ethanol metabolism, from *ADH1 *to *ADH5*. Four of these enzymes, Adh1p, Adh3p, Adh4p and Adh5p reduce acetaldehyde to ethanol during glucose fermentation, while Adh2p catalyzes the reverse reaction of oxidizing ethanol to acetaldehyde. The cytosolic *ADH1 *gene product is the major enzyme that is responsible for converting acetaldehyde to ethanol, and its transcription is repressed when cells are grown on a non-fermentable carbon source such as ethanol or glycerol.

In the present study, we explore the possible influence of a non-*Saccharomyces *yeast such as *Candida stellata *strain, recently reclassified as *Starmerella bombicola *[22], on the fermentation activity of *S. cerevisiae *during mixed fermentation. Trials were carried out using immobilized *S. bombicola *cells in order to confine non-*Saccharomyces *cells and to allow harvesting of *S. cerevisiae *cells, separately. In previous studies, the combined use of *S. cerevisiae *and *S. bombicola *had also been proposed in order to enhance glycerol content and their overall analytical profiles [23-25].

In this context, we evaluated the gene expression and enzymatic activities in *S. cerevisiae *strain as well as the fermentation products of the resulting wines.

## Results and discussion

**Figure 1:**
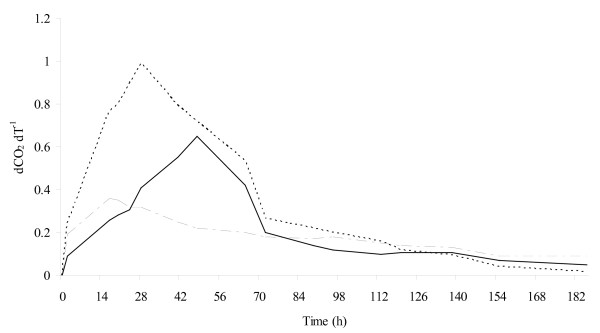
**Fermentation rate**. Fermentation kinetics of pure *S. cerevisiae *culture (continous line), pure *S. bombicola *culture (mixed line) and mixed culture (dashed line). dCO_2_/dT, CO_2 _production rate (grams of CO_2 _evolved per litre per hour). Results are the mean of two independent biological trials, SD was less than 10%.

The evolution of the fermentation process is shown in Figure 1. As expected, the maximum fermentation rate (dCO_2_/dt) of mixed culture was higher than that of *S. cerevisiae *pure culture and it also reached its maximum level faster than *S. cerevisiae *pure culture (0.99 g l^-1 ^h^-1 ^at 28th h, 0.65 g l^-1 ^h^-1 ^at 48th h respectively). Indeed, immobilized cells of *S. bombicola *(10% wet/wt vol^-1^, corresponding to 1.3 × 10^8 ^cell ml^-1^), despite low fermentation activity, increased fermentation rate because of the high concentration of biomass [23]. Cell release from the beads was very low (< 10^3 ^cells ml^-1^), which indicated that the matrix was stable without any interference with *S. cerevisiae *cell harvest.

**Figure 2:**
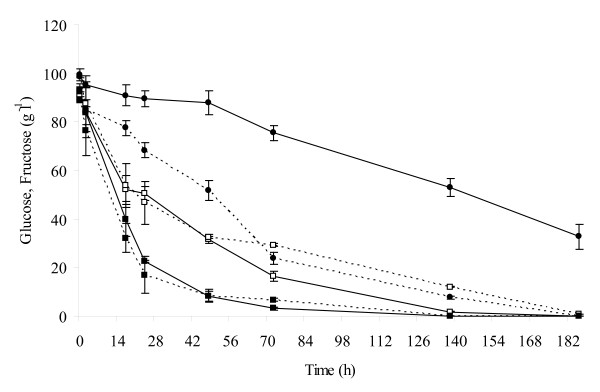
**Consumption of sugars**. Progress of glucose (continuous lines) and fructose (dashed lines) consumption throughout fermentation in mixed culture (■), in *S. cerevisiae *pure culture (□) and in immobilized *S. bombicola *pure culture (●).

One of the most important features during wine production is the total consumption of sugar. Pure culture of *S. cerevisiae *consumed glucose and fructose at almost the same rate only during the first 48 hours of fermentation (Figure 2). After that, *S. cerevisiae *pure culture began to consume glucose faster than fructose determining the total use of sugars at 186th h (glucophilic yeast). Immobilized cells of fructophilic yeast *S. bombicola *[23], showed opposite behaviour consuming fructose faster than glucose (Figure 2). In fact, fructose was completely consumed at 186^th ^h, while glucose was consumed slowly. Mixed culture fermentation showed that the combined use of *S. cerevisiae *and immobilized *S. bombicola *cells leads to contemporary, fast and complete consumption of both sugars (at 138th h).

**Figure 3:**
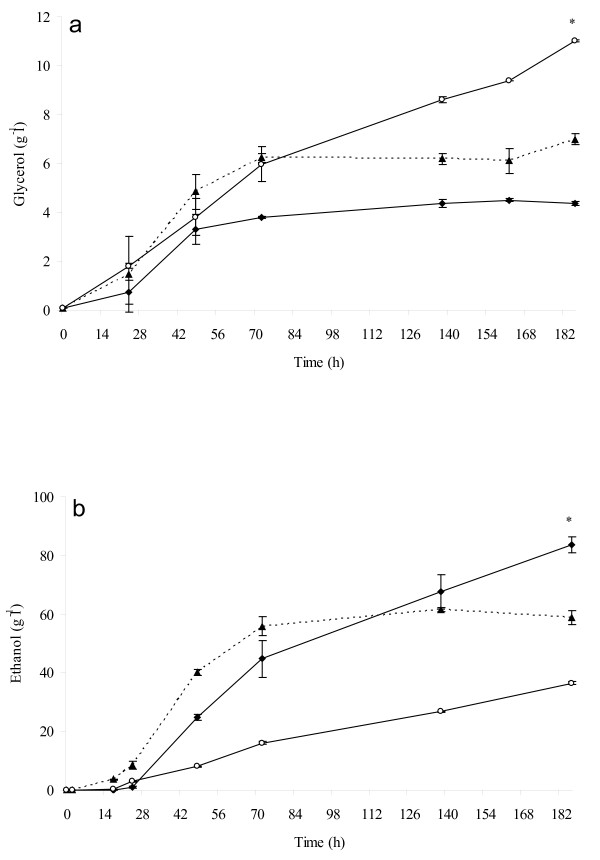
**Glycerol and ethanol production**. Evolution of glycerol (a) and ethanol (b) during fermentation carried out by mixed culture (▲ dashed lines), pure *S. cerevisiae *culture (♦ continuous line) and immobilized *S. bombicola *pure culture (○ continuous lines). The data represented are the mean of three technical repetitions for two independent biological samples ± SD. Asterisk represents significantly different values according to the Duncan test (0.05%).

**Figure 4:**
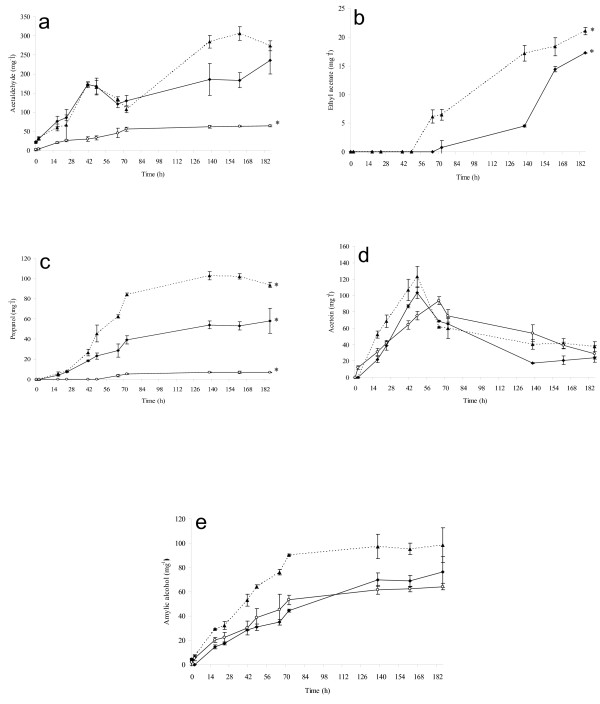
**Fermentation by-products**. By products produced during wine fermentation in mixed culture (▲ dashed lines), in *S. cerevisiae *pure culture (♦continuous lines) and in immobilized *S. bombicola *pure culture (○ continuous lines). Production of (a) acetaldehyde, (b) ethyl acetate, (c) propanol, (d) acetoin and (e) amylic alcohols. The data represented are the mean of the three technical repetitions for two independent biological samples ± SD. Values displaying asterisk are significantly different according to the Duncan test (0.05%).

As expected, pure culture of *S. bombicola *produced high quantity (11 g l^-1^) of glycerol, confirming previous results [24,25] (Figure 3a). Mixed culture produced glycerol faster and higher than *S. cerevisiae *pure culture (7.0 g l^-1^, 4.4 g l^-1 ^respectively) indicating that immobilized *S. bombicola *cells positivel*y *affect the final amounts of glycerol. Evolution of ethanol showed different kinetics in mixed and pure fermentations (Figure 3b). Immobilized cells of *S. bombicola *in pure culture produced the smallest amount of ethanol (36.5 g l^-1^). During the first 72 h of fermentation, mixed culture produced ethanol faster and in higher concentration than that exhibited by *S. cerevisiae *pure culture, remaining stable until the end of fermentation (58.9 g l^-1^). Pure culture of *S. cerevisiae *showed a lower trend of ethanol production, but at the end of fermentation, its concentration was higher than that exhibited by mixed culture (83.6 g l^-1^). This is a very interesting behavior since the reduction of final ethanol concentration in winemaking is one of the most investigated topics [26-28]. The principal by-products in mixed fermentation were produced mainly due to the metabolic activity of *S. cerevisiae *strain (Figure 4). Actually, the trend of these compounds is closely related to that showed by *S. cerevisiae *pure culture, while immobilized *S. bombicola *cells showed a significantly lower production of acetaldehyde, ethyl acetate and n-propanol (Figures 4a, b and 4c). Mixed fermentation exhibited a significantly higher amount of n-propanol and ethyl acetate (Figures 4b and 4c) even if the final concentration turned out to be far from the sensory threshold level. As regards acetic acid production, we noted that there was a lower level of acetic acid in the mixed culture than that in the pure culture of *S. cerevisiae *(0.82 g l^-1^, 0.94 g l^-1 ^respectively), while the pure culture of *S. bombicola *immobilized cells produced only a small amount of acetic acid (0.23 g l^-1^).

The overall results of the metabolic interactions between *S. cerevisiae *and *S. bombicola *immobilized cells in mixed culture confirm their strong influence on fermentation rate, glycerol, ethanol production and utilization of sugars. At the same time, a reduced influence on the main by-products was also seen. In this context, we investigated the influence of *S. bombicola *on the alcoholic fermentation of *S. cerevisiae *evaluating pyruvate decarboxylase and alcohol deydrogenase gene expression and activity. These are two key enzymes of the alcoholic fermentation process. Pyruvate decarboxylase irreversibly converts pyruvate into acetaldehyde and CO_2 _, and its gene transcription is higher in cells grown on glucose than that grown on ethanol [29-36], while alcohol deydrogenase 1 is the main cytosolic enzyme involved in the formation of ethanol during glycolysis [37].

**Figure 5:**
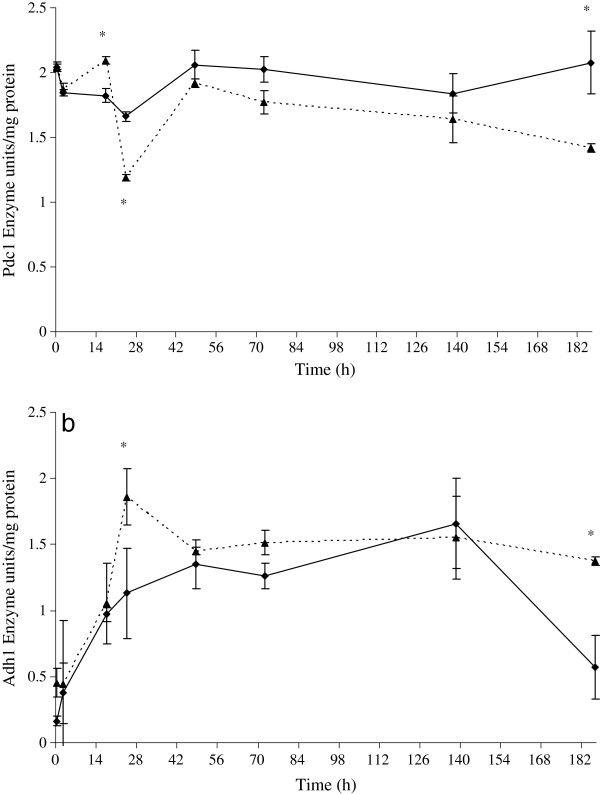
**Enzymatic activity**. Enzymatic activity of Pdc1 (a) and Adh1 (b) in *S. cerevisiae *at different stages of fermentation. Dashed lines represent mixed culture, and continuous lines, pure *S. cerevisiae *culture. Error bars show standard deviations including three technical repetitions for two independent biological samples. Values displaying asterisk are significantly different according to the Duncan test (0.05%).

The activity trends of Pdc1 and Adh1 of *S. cerevisiae *during fermentation in pure and mixed cultures are shown in Figures 5a and 5b. Pdc1 enzyme in mixed fermentation at 17th h showed maximum activity (Figyre. 5a). After this time, the activity of Pdc1 in mixed culture decreased quickly (24th h), then rose again remaining stable and low in comparison with pure culture (Figure 5.5b). On the other hand, the behavior of enzymatic activity in pure culture trial was quite stable during the first stage of fermentation.

Adh1 activity of *S. cerevisiae *in mixed fermentation was higher than that exhibited by pure culture during the whole fermentation process. After 24 h, it reached its maximum level (72% greater than pure culture) and, after a decrease, remained stable until the end of the fermentation process. In pure culture, there was a progressive increase until the maximum level at 138th h and a notable decrease at the end point (81% lower than in mixed culture). Interestingly, there was an opposite trend of these two enzymes in mixed fermentation, i.e., when Adh1 activity was at its peak, Pdc1 activity was at its minimum.

Since both *ADH1 *and *PDC1 *genes encode these two enzymes that catalyze the synthesis of ethanol from pyruvate during alcoholic fermentation, they were expected to be highly expressed under wine fermentation conditions [38,39].

**Figure 6:**
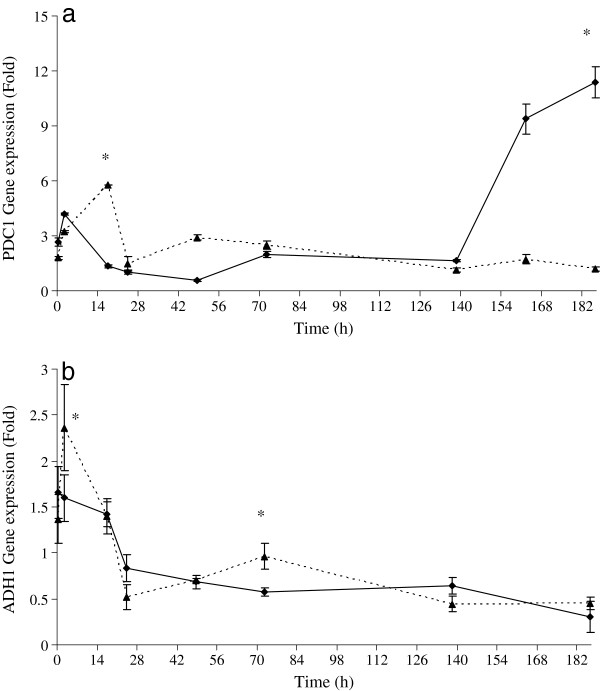
**Gene expression**. Expression levels of *PDC1 *(a) and *ADH1 *(b) in *S. cerevisiae *at different stages of fermentation. Dashed lines represent mixed culture, and continuous lines, pure *S. cerevisiae *culture. Error bars show standard deviations, including three technical repetitions for two independent biological samples. Relative normalized fold expression is calculated using *TAF10 *as reference gene. Values displaying asterisk are significantly different according to the Duncan test (0.05%).

Results of gene expression are showed in Figure 6 where, during the first 72 h, *PDC1 *expression in mixed culture was higher than that exhibited by pure culture reaching its maximum level at 17th h (5.8 and 1.4 folds, respectively). Subsequently, the expression of *PDC1 *in mixed culture was stable until the end of fermentation, while in pure culture, a fast enhancement during the final step of fermentation (11.3 folds) was seen. This behavior could be explained by the presence of fructose (ca. 12 g l^-1^) in this last stage of fermentation (Figure 2) that determined an improvement in gene transcription level. Similar results were reported by Molina and co-workers [40] where, at 60-80% of sugar consumption, a peak of *PDC1 *expression in synthetic must was noted.

*S. cerevisiae ADH1 *for both pure and mixed fermentation was highly induced at the initial step. At the 2nd h, the expression level of this gene quickly increased in mixed fermentation, and it was higher than that seen in pure culture (2.36 and 1.52 folds, respectively). This maximum expression level was followed by a rapid down regulation during the following 22 hours (from 2.36 to 0.52 folds). Furthermore, in previous studies, it has been reported that the biosynthesis of Adh1 takes place basically during the adaptation of the yeasts to the musts (first 4 h) [41,42]. Subsequently, this gene in mixed culture slightly increased the expression level until 72th, to remain constant at 0.44 fold. Similarly, *ADH1 *expression of pure culture decreased in the first 24 hours and after which it remained stable towards the end of fermentation (0.3 fold).

Similar expression profiles observed for *PDC1 *and *ADH1 *strongly support the hypothesis that the enzymes Pdc1 and Adh1 might be co-regulated and participate in the same metabolic pathway [40].

Present results indicate that the activity and gene expression of pyruvate decarboxylase and alcohol deydrogenase in *S. cerevisiae *are influenced in mixed culture by *S. bombicola*. The metabolic interactions in mixed yeast culture of *S. bombicola *immobilized cells and *S. cerevisiae *under winemaking conditions are complex that lead the modification of the kinetics of fermentation and analytical profile of wine, influencing the activity and gene expression of pyruvate decarboxilase and alcohol deydrogenase.

## Conclusions

In mixed fermentation, *S. bombicola *immobilized cells greatly influenced the fermentation behaviour of *S. cerevisiae *and the analytical composition of wine. The influence of *S. bombicola *seems to be very complex and not limited to a synergistic or additive effect on the analytical profile of wines. Metabolic modifications in *S. cerevisiae *alcoholic fermentation were observed since significant modification of alcohol dehydrogenase and pyruvate decarboxylase gene expression and enzymatic activity was exhibited. Metabolic modifications in *S. cerevisiae *coupled with *S. bombicola *immobilized cells showed an enhancement of both pyruvate decarboxylase activity and gene expression during the first stage of fermentation (2-17 h). A similar behavior was shown for alcohol dehydrogenase gene expression, and enzymatic activity also exhibited the same enhancement but with a delay of 7 hours (at 24th h).

This study is the first attempt to investigate yeast metabolic interaction which monitors gene expression and enzymatic activity during controlled mixed fermentation in winemaking.

## Methods

### Microorganisms

Yeast strains used in this work were commercial strain Lalvin EC1118 *Saccharomyces cerevisiae *and *Starmerella bombicola *(formerly *Candida stellata*) DBVPG 3827 strain coming from the Industrial Yeasts Collection of the Dipartimento di Biologia Vegetale, University of Perugia (DBVPG).

### Media

Synthetic grape juice (SGJ) was used in fermentation tests. Each litre of SGJ was composed of three different solutions: solution A (500 ml), solution B (250 ml), and solution C (250 ml). The composition of SGJ was as follows (per litre): solution A: _D_-glucose, 100 g; _D_-fructose, 100 g; ergosterol, 10 mg; Tween80, 1 ml; solution B: L-(+)-tartaric acid, 6.0 g; L-(-)-malic acid, 2.0 g; citric acid, 0.5 g; solution C: YNB (yeast nitrogen base without amino acids and ammonium sulfate) (Difco), 1.7 g; CAA (vitamin-free Casamino Acids) (Difco), 2.0 g; CaCl_2_, 0.2 g; arginine-HCl, 0.8 g; L-(2)-proline, 1.0 g; L-(2)-tryptophan, 0.1 g. Solutions B and C were buffered at pH 3.5 with NH_4_OH and H_3_PO_4_, respectively. Four millilitres of Ergosterol stock solution (Tween 80, 6.25 ml; Ergosterol, 62.5 mg in ethanol to make 25 ml) was added to the glucose-fructose solution to complete solution A. All three solutions were sterilized at 121°C for 20 min separately and then combined aseptically. YPD medium (2% glucose, 2% peptone and 1% yeast extract; all w/v) was used to produce biomass for the immobilization system.

### Fermentation conditions and sampling

In order to investigate the influence of immobilized *S. bombicola *on *S. cerevisiae *fermentation activity, fermentations in mixed cultures were set up together with pure culture fermentations of both strains as control. The basic principle of immobilization consists in keeping *S. bombicola *cells separate from free *S. cerevisiae *cells which can be sampled and analyzed separately. Metabolites from both microorganisms are allowed to pass through the substrate even if two different yeasts grow separately. Duplicate fermentations were carried out in 1-litre glass minifermentors (containing 500 ml of SGJ under static conditions at 25°C) with two ports, one for gas flow and the other for an inoculum of beads, and a septum of frit glass in order to maintain the beads in the medium and to allow carbon dioxide to come out. Cells for immobilization were grown in YPD at 25°C in a rotary shaker (150 rpm) for 72 h (*S. bombicola*), harvested by centrifugation, washed three times with sterile distilled water and added to 2.5% Na-alginate (Carlo Erba, Milan, Italy) at a ratio of 5% (wet weight vol^-1^) (biomass moisture, 70%; final concentration, 1.3 × 10^9 ^cells per g of beads). By means of a peristaltic pump, this mixture was then dripped into CaCl_2 _(0.1 M) to induce gelation. After 1 h, the beads were washed several times with sterile distilled water and used immediately. The inoculum for immobilized cells of *S. bombicola *was 10% (wet weight/vol) of the amount of beads in the medium (corresponding to 1.3 × 10^8 ^cells per ml). Yeast culture of *S. cerevisiae *was pre-incubated in SGJ at 25°C in a rotary shaker (150 rpm) for 48 h, harvested by centrifugation, washed with sterile distilled water, and the procedure was standardized to provide an inoculation level of 10^6 ^cells/ml. Before the inoculum and, at the end of fermentation in mixed and pure culture, 10 g of beads were maintained under agitation in 100 ml 1% Na-citrate solution (w/v) for 1 h to release the cells, and cell viability was evaluated by the standard plate count techniques in Lysine medium. The evolution of fermentations was evaluated gravimetrically by weight loss due to the carbon dioxide evolved. Samples of the culture medium were taken at different stages of fermentation from each minifermentor. One part of all the samples was used to determine cell number by light microscopy using a Thoma-Zeiss counting chamber, and optical density was measured at 600 nm (OD_600_). The other part of each sample was centrifuged for 5 min at 2000 g. Supernatants were filtered through a 15 mm syringe filter Phenex (0.2 μm pore diameter, Phenomenex, Torrence CA, USA), stored at -20°C and analyzed later to determine residual sugars, ethanol, glycerol, acetaldehyde, ethyl acetate, propanol, acetoin and amylic and iso-amylic alcohols concentration. The cell pellet for RNA and protein extraction was mixed with glycerol and conserved at -80°C until use.

### Analytical determinations

Ethanol was measured by a gas-liquid chromatography (GLC) analysis [43]. Acetaldehyde, ethyl acetate, acetoin and higher alcohols were determined by direct injection into the GLC system. The samples were injected into a 30 m by 0.32 mm, 0.25 μm film thickness column Zebron ZB-WAXPlus (Phenomenex, Torrance, California, USA) with an internal standard of 1-penthanol (162 mg l^-1^). Nitrogen was used as the carried gas. A Shimadzu gas chromatograph (Japan), equipped with a flame ionization detector, was used. The oven temperature ranged from 40°C to 200°C. The temperature of the injector and the detector was 150°C.

Glucose, fructose (kit no. 139106), and glycerol concentration (kit no. 148270) were determined by using specific enzymatic kits (Boehringer, Mannheim, Germany). Volatile acidity (expressed as grams of acetic acid per litre) was quantified by steam distillation according to the official analytical methods [44].

### RNA isolation and cDNA synthesis

Total RNA was extracted using the NucleoSpin RNAII kit (Macherey-Nagel, Düren, Germany) from samples containing approximately 10^7 ^cells following the protocol provided by the manufacturer. RNA concentration was determined using Nanodrop ND 1000 (Thermo Fisher scientific, Wilmington, DE, USA), and RNA quality was tested by electrophoresis on 1.5% agarose gel.

cDNA was synthesised from the isolated RNA by the Maxima First Strand cDNA Synthesis kit for RT-qPCR (Fermentas) as recommended by the manufacturer using oligo (dT)_18 _and random hexamer primers to prime synthesis of first strand cDNA.

### Primer design

In order to identify the housekeeping and target genes of *S. cerevisiae *coding for selected proteins, we used the Genome database available at NCBI (http://www.ncbi.nlm.nih.gov). *TAF10 *gene (RNA Pol II transcription factor activity/transcription initiation and chromatin modification) was used as housekeeping reference because it turned out to be one of the genes whose expression remained stable, independent of the growth conditions as highlighted by Teste and co-workers [45].

Real-time PCR primers were designed using the OligoAnalyzer 3.0 software (available at http://eu.idtdna.com/analyzer/) and synthesised by MWG Biotech (MWG Biotech, Germany). Annealing temperature of all primer pairs was 60°C. Amplification efficiency was determined by the serial dilution method beginning from a cDNA pool [46], and PCR efficiency was calculated by the equation E = 10[-1/slope]. Each primer was tested on agarose gel to verify its specificity. Table 1 shows the primer sequences used in this study along with their amplicon sizes, and their resulting efficiencies. Primers have been checked for absence of cross-amplification using *S. bombicola *cDNA as template.

**Table 1:** PCR primers used in this study

Gene	Sequence (5'-3')	Amplicon length (bp)	Primer efficiency
*ADH1*	ATCCAACTGTCCTCACGCTGACTT	104	1.01
			
	TACCTTGAGGAATGTGAGCGGCTT		

*PDC1*	TGTCGAATTCCACTCCGACCACAT	114	1.00
			
	TAACCCTTAGCGGCGTCAGCAATA		

*TAF10*	GCAGCTATTGCAAGGACAGCAACA	142	1.08
			
	ATTGAGCCCGTATTCAGCAACAGC		

### Gene expression analysis by real time PCR

The expression levels were determined using real time PCR. All real-time reactions were performed using Mastercycler^® ^ep realplex (Eppendorf, Hamburg, Germany). A ready-to-use RealMasterMix SYBR ROX 2.5X (5 Prime, Hamburg, Germany) was used according to the manufacturer's instructions. Reactions were performed in 150 μl twin.tec real time PCR plates 96 (Eppendorf), and each 10 μl reaction mixture contained: 200 nM of each primer, 4.5 μl of 2.5X RealMasterMix SYBR ROX, 4 μl of cDNA and H_2_O to reach a final volume. All real time PCR experiments were carried out using two biological repetitions, and the samples were considered in triplicate. A negative control without a cDNA template was included to ensure that the samples did not have any unspecific SYBR Green fluorescence. The program used was the follow: 95°C for 2 min; 40 cycles of 95°C for 20 s, 20 s at 55°C, 68°C for 30 s; 1 cycle of 68°C for 1 min. After this, melt curve data were then collected. Gene expression levels are shown as the concentration of the studied gene normalized with the concentration of the housekeeping *TAF10 *gene.

### Enzyme assay

Whole-cell homogenates were prepared following the method by Blumer and co-workers [47]. Protein concentrations of cell extracts were determined by the Lowry method with a bovine serum albumin (Sigma) as the standard. Enzyme assays were performed at 25°C with a Shimadzu UV1800 spectrophotometer at 340 nm. Reaction rates were linearly proportional to the amount of cell extract added. One unit (U) is defined as the amount of enzyme catalyzing the conversion of one micromole of substrate min^-1^. Specific activity is expressed as U per mg of protein. The assay mixture for each enzyme is described below.

Alcohol dehydrogenase (NAD-dependent, ADH1 EC.1.1.1.1). The assay mixture contained 60 mM sodium pyrophosphate buffer (pH 8.5), 100 mM ethanol and 50 mM NAD^+^. The reaction was started with a cell extract.

Pyruvate decaroxylase (PDC1 EC.4.1.1.1). The reaction mixture contained 40 mM imidazole HCl buffer pH (6.5), 5 mM MgCl_2_, 0.2 mM thiamine pyrophosphate (TPP), 0.15 mM NADH, 88 U of alcohol dehydrogenase and a cell extract. The reaction was started with 50 mM pyruvate.

### Statistical analysis

Analysis of variance (ANOVA) was applied to the experimental data for the by-products evaluated during pure and mixed fermentations. The means were analyzed using the SuperANOVA software, version 1.1, for Mac OS 9.1. The significant differences were determined by means of the Duncan test, and the results were considered significant if the associated P values were below 0.05.

## Competing interests

The authors declare that they have no competing interests.

## Authors' contributions

VM, LO, MC and FC contributed equally to this manuscript. All authors participated in the design and discussion of the research. VM and LO carried out the experimental part of the work. VM, LO, MC and FC carried out the analysis of the data and wrote the manuscript. All authors have read and approved the final manuscript.
